# An overview on the treatment and outcome factors of ankle fractures in elderly men and women aged 80 and over: a systematic review

**DOI:** 10.1007/s00402-021-04161-y

**Published:** 2021-09-21

**Authors:** Marieke S. van Halsema, Rick A. R. Boers, Vincent J. M. Leferink

**Affiliations:** Radboud University Medical Centre, Nijmegen, The Netherlands

**Keywords:** Aged 80 and over, Ankle fracture, Surgical treatment, Conservative treatment, Weight-bearing

## Abstract

**Introduction:**

This article is a systematic review of the literature on elderly aged 80 and over with an ankle fracture. Low energy trauma fractures are a major public health burden in developed countries that have aged populations. Ankle fractures are the third most common fractures after hip and wrist fractures. The purpose of this review is to provide an overview of the treatments and the used outcome factors.

**Methods:**

PubMed, Embase, Cochrane Library, and CINAHL were searched to retrieve relevant studies. Studies published in English or Dutch concerning the treatment of ankle fractures in patients aged 80 and over were included.

**Results:**

Initially 2054 studies were found in the databases. After removing duplicate entries, 1182 remained. Finally, after screening six studies were included, of which three cohorts studies and three case series. Six different treatments were identified and described; ORIF, transarticular Steinmann pin, plaster cast with or without weight-bearing, Gallagher nail and the TCC nail. Furthermore, 32 outcome factors were identified.

**Discussion:**

The various studies show that practitioners are careful with early weight-bearing. However, if we look closely to the results and other literature, this seems not necessary and it could potentially be of great value to implement early weight-bearing in the treatment. Furthermore, quality of life seems underreported in this research field.

**Conclusions:**

ORIF with plaster cast and permissive weight-bearing should be considered for this population since it seems to be a safe possibility for a majority of the relatively healthy patients aged 80 and over. In cases where surgery is contra-indicated and a plaster cast is the choice of treatment, early weight-bearing seems to have a positive influence on the outcome in the very old patient.

## Introduction

Low energy trauma fractures among the elderly are a significant and growing concern for public health in developed countries. The annual incidence of ankle fractures is reported to be around 150 out of 100,000 people [1, 2]. In 1970, the incidence was reported to be 57 out of 100,000 people and it is expected that it will increase to approximately 270 out of 100,000 in 2030. Low energy trauma is the main cause for this type of fracture. The incidence is highest in women and with aging, the incidence increases more in women (0.3% per year) than in men (0.1% per year) [1, 3]. Ankle fractures have a bimodal age distribution, with one peak in young men and one peak in elderly women around the age of 60 years [1, 4]. It is a challenge to treat these fractures in elderly people because they are prone to complications like infection and loosening of osteosynthetic material, and the outcome of these injuries is less predictable compared to the outcome in younger patients [4–6].

In literature regarding hip fractures, a difference between elderly men and women has been described before. Women display a faster decline of bone mineral density (BMD) compared to men, and this seems to be a major contributing factor. Additionally, it seems that women with hip fractures have lower values of endocortical width and cortical thickness than men [7, 8].

Literature on ankle fractures and its involving factors is scarce, especially those concerning the difference between elderly men and women and in patients aged over 80. With an aging population, it is important to understand how to manage this injury best to achieve the best outcome possible [9].

To answer the question how to manage this injury best in the very old, we tried to find out which method of treatment is most effective. Therefore, the goal is to identify which kind of outcome factors are used in this research field. Having first considered this, we will be able to define which outcome factor, or combination of outcome factors, makes a treatment suitable for this population.

Furthermore this literature study aims to provide an overview of the different kinds of treatments for elderly men and women aged 80 and over with an ankle fracture, and which treatment is most effective for the target population.

## Methods

### Rationale of study design and study population

This systematic review was conducted following the PRISMA guidelines [10]. The study population is patients with ankle fractures aged 80 years and over. The cut-off age of 80 years was chosen because in the aging countries people are more and more active at a higher age. Retirement at 67 years or at an even higher age is in discussion and in some countries reality. At the same time elderly people are stimulated to continue living in their own homes as independently as possible. We wanted to discuss treatment of ankle fractures in the elderly who are more or less prone to inactivity, in relation to being active and living on your own.

### Search strategy

A comprehensive electronic search was performed on the 25th of June 2020 by author (MH) in Pubmed, Cochrane Library, EMBASE, CINAHL to obtain all publications on studies relating to ankle fractures in elderly aged 80 + , between January 2010 and June 2020. An expert librarian at Radboud University was consulted to guide the search through the medical library index. The search strategy was made in a way that nearly every article with our age group will be found. We used different kinds of studies to identify age-specific and geriatric search filters to make the search strategy as sensitive as possible [11, 12]. Additionally, articles that focus on more age groups than just our age group, are not excluded by the search strategy. The only studies that are excluded, are studies that do not name the group of the very old, or a synonym, in their title, abstract nor in their subject headings. The articles that are found with our strategy could have multiple age populations, but will always contain the population 80 years and older. Search strings for the various databases are provided in Appendix 1.

### Eligibility criteria

The most important inclusion criteria were that patients should be aged 80 and over, they should have an ankle fracture, the study must be about the treatment. The included studies must have been published after 2010. We made a considered decision concerning the inclusion criteria of the year cut-off. In a quickly changing world, especially for elderly people, it is opportune to check recent literature. In the year 2000 for example, the situation was really different when you look at housing, help, independency of institutions, selfsupportingness, etcetera. The most important exclusion criteria were multi trauma or high-energy trauma patients, pathologic fractures and fractures that are not ankle luxation fractures like. Pilon fractures were excluded and were defined based on the description of the study. Pilon fractures are comminuted intraarticular distal tibia and fibular fractures, with loss of length and stability of the tibia, most of them resulting from a high-energy trauma, like fall from height. Pathologic fractures were defined as fractures as a results of cancer or complications of diabetes like a charcot foot. Fractures resulting from osteoporosis were included in the study. Eligibility criteria are displayed in Table 1. In case of insufficient high-quality studies, case series will be included.

**Table 1:** Eligibility criteria

Inclusion criteria	Exclusion criteria
Elderly people aged 80 and over	Articles from before 2010
Study should be about the treatment	Multi trauma patients
The patient should have one of the following ankle fractures:	High energy trauma
Lateral malleolus fracture	Pathologic fractures
Medial malleolus fracture	Expert opinions, case reports, cross-sectional studies, systematic reviews, meta-analysis, cadavers studies
Bimalleolar ankle fracture	Fractures that are not ankle luxation fractures like:
Bimalleolar equivalent fracture	Pilon tibial fracture
Trimalleolar fracture	Isolated fibula shaft fracture (without involvement of the ankle joint)
Posterior malleolus fracture	Lower leg fractures
Maisonneuve fracture	Distal tibia shaft fracture
Distal tibia fracture	Studies about multiple fracture sites
Distal fibula fracture	
It has to be a clinical research	
The article should be written in English or Dutch	

### Study selection

Study selection was completed by two independent reviewers (MH and RB). In the first phase, the title and abstract were screened to identify studies that could be eligible for inclusion. In the second phase, the full-text of those studies were assessed for eligibility by both reviewers. Additionally, the reference list of the included studies was explored to identify additional potential articles for inclusion. Any disagreement between the two reviewers was resolved during a consensus meeting and if necessary, a third reviewer (VL) was involved. Rayyan Qatar Computing Research Institute (QCRI), the systematic review web app, was used for the screening process [13].

### Data extraction and analysis

Data extraction was performed by one author (MH) and checked by the second author (RB). From the included studies, the following data were collected: authors, study type, year of publication, the total number of participants, gender, mean age, number of patients aged 80 and over, fracture type, treatments, outcome factors, and follow-up.

### Quality assessment

Two reviewers (MH, RB) independently performed a quality assessment of the included articles. For randomized controlled trials, the Cochrane Risk of bias (2.0) (RoB2) will be used. For non-randomized studies (cohort or case–control), the Newcastle Ottawa Scale (NOS) will be used [14]. For case series, JBI critical appraisal checklist for case series will be used [15, 16]. Differences in scores between the reviewers were resolved during a consensus meeting.

## Results

### Literature selection

**Fig. 1:**
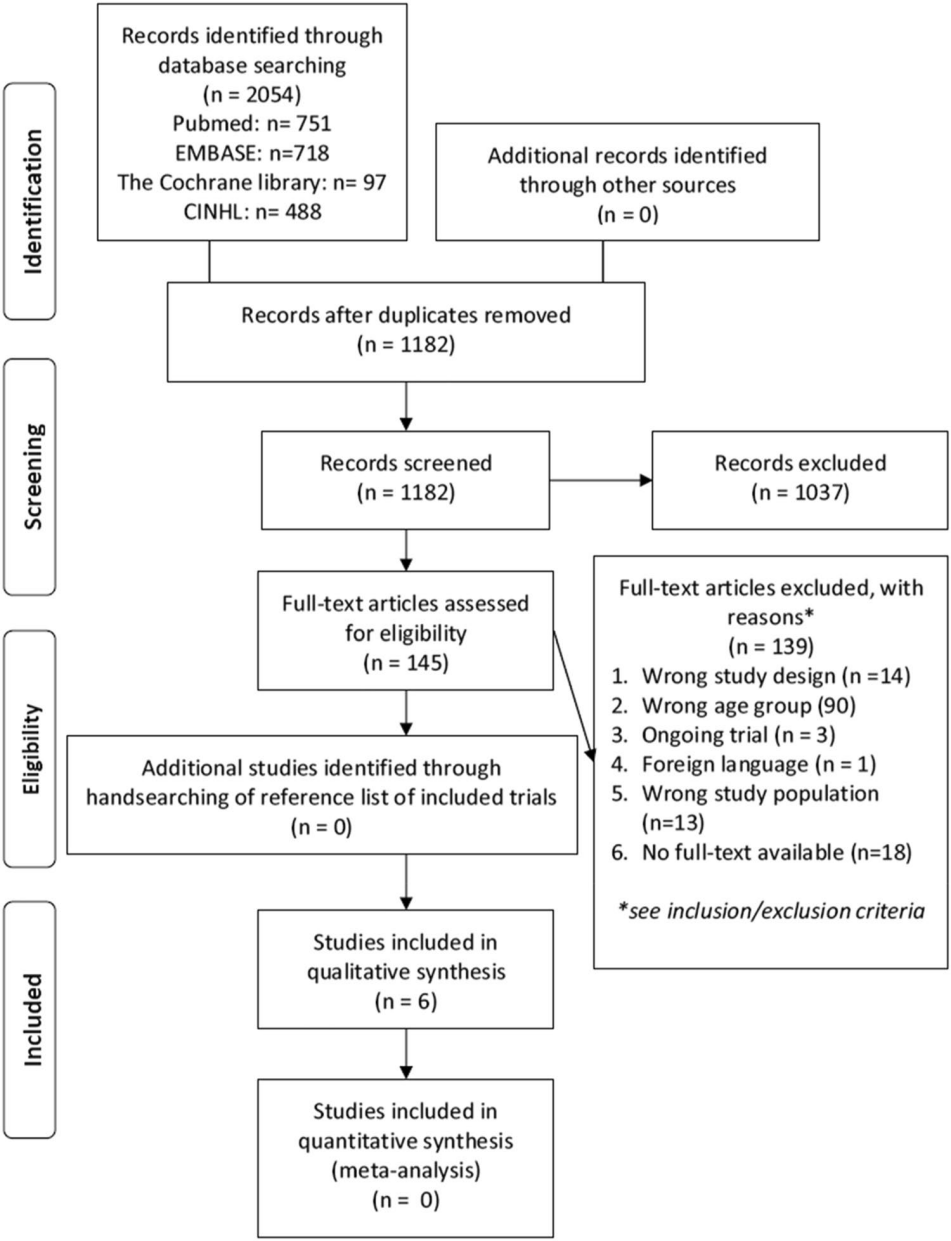
PRISMA flowchart of screening process

Initially, 2054 studies were searched from databases. After removing duplicate entries, 1182 remained. In the first phase, after reading the title and abstract, 1037 studies were excluded. After reading the full-text in the second phase, 6 studies were included. A flow chart of the study screening process is shown in Fig. 1.

### Characteristics of eligible studies

All studies included were published between January 2010 and June 2020 (Table 2). Initially, case series were an exclusion criteria. However, insufficient high-quality evidence led to the decision to include case series. Therefore, three cohort studies and three case series were included. In total six studies with 264 patients, concerning 265 ankles were included in the review. Of these patients, at least 184 met the inclusion criteria and were aged 80 and over. Overall, of the 264 patients the age ranged between 48 and 101. The age of the 184 included patients ranged between 80 and 101. In total six different treatments were discussed.

**Table 2:** Characteristics of included studies

References	Study type	Participants (N), sex (M/F)	Mean age in years (range)	Number of patients aged over 80 (M/F)	Fracture type (N)	Treatments (N)	Follow-up time
Meijer et al. [21]	Single-center retrospective cohort study	15 (5/10)	82 (61–96)	At least 9 (2/7)	*Transarticular Steinmann pin/External fixation* Weber type B (8/3) Weber type C (1/3) Bimalleolar/Tri- (4/5)	Steinmann pin (9) External fixation (6)	No specific information about the follow-up time
Schray et al. [20]	Single-center retrospective observational cohort study	58	Mean age 77.7 (SD 6.2 years)	Could not be determined from the article	Most frequent fracture type (AO type) 44 B (72%)	External fixation converted to ORIF External fixation as final treatment Open reduction and internal fixation	Mean of 16 +/- 8.5 months
Lorente et al. [22]	A multicenter prospective cohort study	70 (31/39)	The mean age was 83 ± 3 years	70 (31/39)	Bimalleolar non-displaced fractures	*Closed cast:* Immobilized patients (control group)(37) Weight-bearing activities (experimental group)(33)	2 years
Shivarathre et al. [19]	Single-center retrospective case series	92 (12/80)	85.2 (80.1–95.1)	92 (12/80)	*Danis-Weber:* Type B (77) Type C (14) Isolated lateral malleolus (4) Isolated medial malleolus (1) Bimalleolar (70) Trimalleolar (16)	*Lateral malleolus* 1/3 Tubular plates (83) DCP (4) Locking plates (4) *Medial malleolus* Two screws (45) One screw (5) Tension band wiring (25) *Posterior malleolus* Anterior–posterior screw fixation (4)	Average of 15 months (range, 9 to 28)
O'Daly et al. [23]	Single-center retrospective case series	9 (1/8)	80.1 (60–101)	6 (1/5)	Bimalleolar fractures (5) Trimalleolar fracture (3) Fracture dislocation of the ankle (1)	Gallagher nail fixation	Mean of 34 months
Armstrong et al. [24]	Single-center retrospective case series	21 ankle in 20 participants (3/17)	76 (48–98)	7	Gustilo–Anderson grade 3B open ankle fracture	Tibiotalocalcaneal nail fixation (total/our population) T2 ankle arthrodesis nail (10/1) Versanail (9/5) Titanium cannulated hindfoot arthrodesis nail (2/1)	The duration of follow-up is short-term. No specific number is stated

### Literature quality

The NOS was used for the assessment of the cohort studies. Studies with a score between 7 and 9 are defined as high quality, 4–6 as moderate quality, and 0–3 as low quality [17]. Among the cohort studies (see Table 3), two were of high quality, and one was of moderate quality. For the case series (Table 4) the JBI checklist was used. Among the case series, two were of high quality and one was of low quality. Munn et al. suggest to present the results of the critical appraisal for all questions via a table rather than summarizing with a score [16]. This advice was followed and a short overview is also provided by an overall judgement. Articles were considered as high quality if they scored “yes” for at least 75% of the criteria, moderate if they scored between 50–75%, and low if less than 50% [18].

**Table 3:** Newcastle–Ottawa Quality Assessment Scale results for included cohort studies

Items		Meijer 2017	Schray 2018	Lorente 2020
Selection	Representativeness of the exposed cohort	*	*	*
	Selection of the non-exposed cohort	*		*
	Ascertainment of exposure	*	*	*
	Demonstration that outcome of interest was not present at start of study		*	*
Comparability	Comparability of cohorts on the basis of the design or analysis	*	*	*
Outcome	Assessment of outcome	*	*	*
	Was follow-up long enough for outcomes to occur		*	*
	Adequacy of follow-up of cohorts	*	*	*
***Quality scores***		*Moderate*	*High*	*High*

**Table 4:** Joanna Briggs Institute critical appraisal tool, checklist case series

Criteria	Shivarathre	O’Daly	Armstrong
1. Where there clear criteria for inclusion in the case series	Yes	Unclear	Yes
2. Was the condition measured in a standard, reliable way for all participants included in the case series?	Yes	Yes	Yes
3. Were valid methods used for identification of the condition for all participants included in the case series?	Yes	Yes	Yes
4. Did the case series have consecutive inclusion of participants?	Yes	Unclear	Yes
5. Did the case series have complete inclusion of participants?	Yes	Unclear	No
6. Was there clear reporting of the demographics of the participants in the study?	Yes	No	Yes
7. Was there clear reporting of clinical information of the participants?	Yes	No	Yes
8. Were the outcomes or follow-up results of cases clearly reported?	Yes	Yes	Yes
9. Was there clear reporting of the presenting site(s)/clinic(s) demographic information?	No	No	No
10. Was statistical analysis appropriate?	No	Not applicable	Not applicable
***Quality scores***	*High*	*Low*	*High*

### Treatments

#### Open reduction and internal fixation (ORIF)

Shivarathre [19] reported 92 patients aged over 80 years who underwent ORIF for unstable ankle fractures from January 1988 to August 2007. Stabilization was performed using small fragment plates and screws. All patients were treated in a non-weight-bearing below-knee plaster cast for at least 6 weeks. Patients with diabetes mellitus were immobilized for an additional 4–6 weeks.

Schray [20] analysed 58 patients who underwent ORIF between January 2015 to May 2017, all patients were aged 70 or above. However, an exact number of patients above the age of 80 is not given.

#### Transarticular Steinmann pin

Meijer [21] reported nine patients over a period of 5 years (January 2008 and December 2012) that met the inclusion criteria. They were treated with closed reduction and internal fixation using a transarticular Steinmann pin and additionally patients were mobilized in a cast. Due to the age restriction, only the Steinmann pin of this study was used in the analyses, the external fixators were left out. The mean age in the Steinmann pin group was 86 years (range 82–88).

#### Plaster cast: early versus non weight-bearing

Lorente [22] analysed 70 patients aged above 80 years treated with a closed cast over a period from 2014 until 2018. In patients who are not fit enough for surgery, the standard procedure was immobilization with a plaster for 6–8 weeks. The study compared weight-bearing versus non weight-bearing.

#### Gallagher nail

**Fig. 2:**
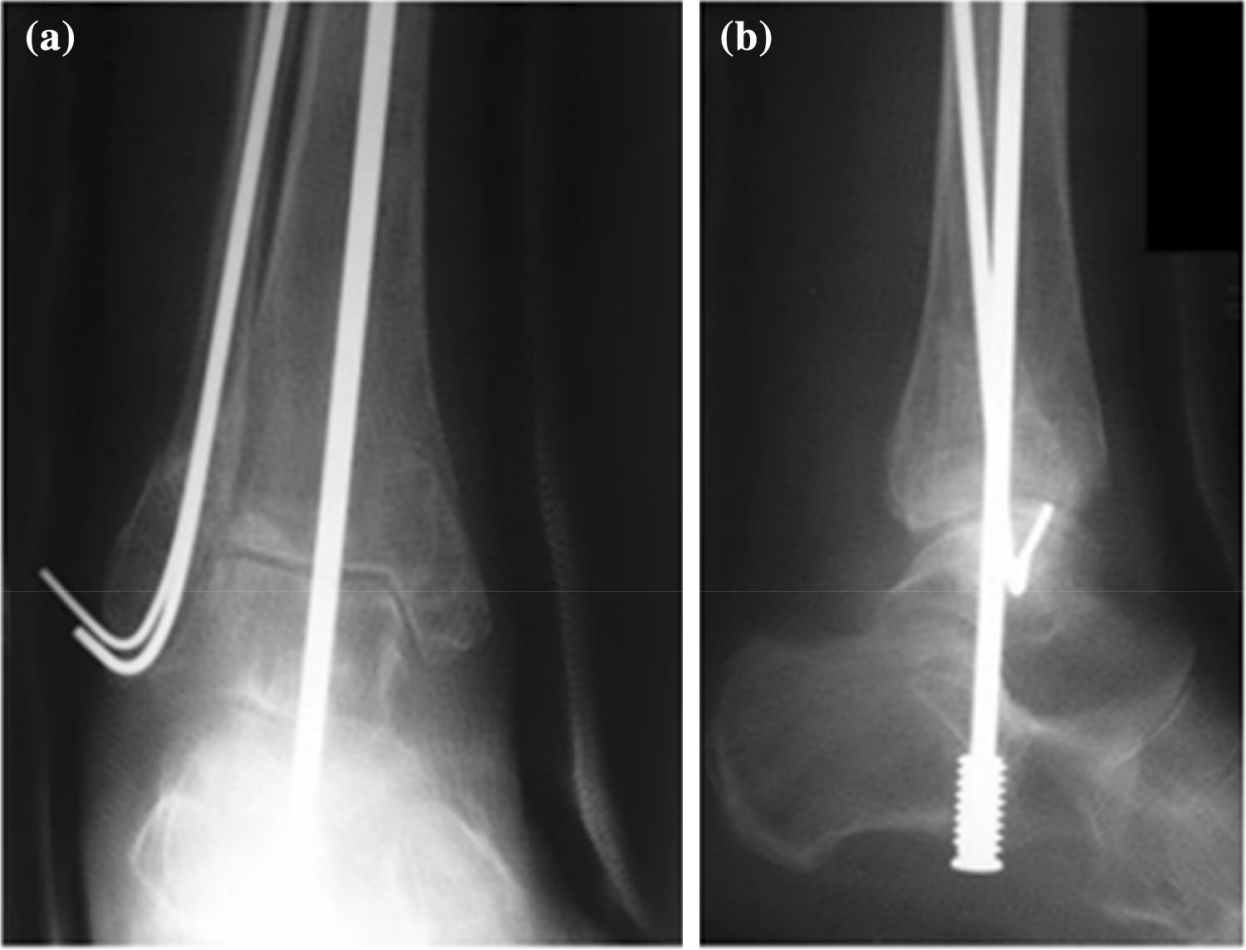
Post-operative **a** AP X-ray and **b** Lateral X-ray ankle: Mortice reduction maintained at 6 weeks post-operatively following Gallagher nail insertion. Kirschner wires have been introduced into the lateral malleolus to stabilize the reduction. Source: Obtained from Ref. 23

O’Daly [23] described nine cases over a 10-year period (1996–2005) of fragility fractures who had failed after closed manipulation of the ankle fracture and in whom the local or general condition precluded internal fixation. Patients were treated with a Gallagher nail which is a modification of the Steinmann pin technique (see Fig. 2). Of these patients, six met our inclusion criteria with a mean age of 87 years (80–101).

#### Tibiotalocalcaneal nail fixation (TCC)

**Fig. 3:**
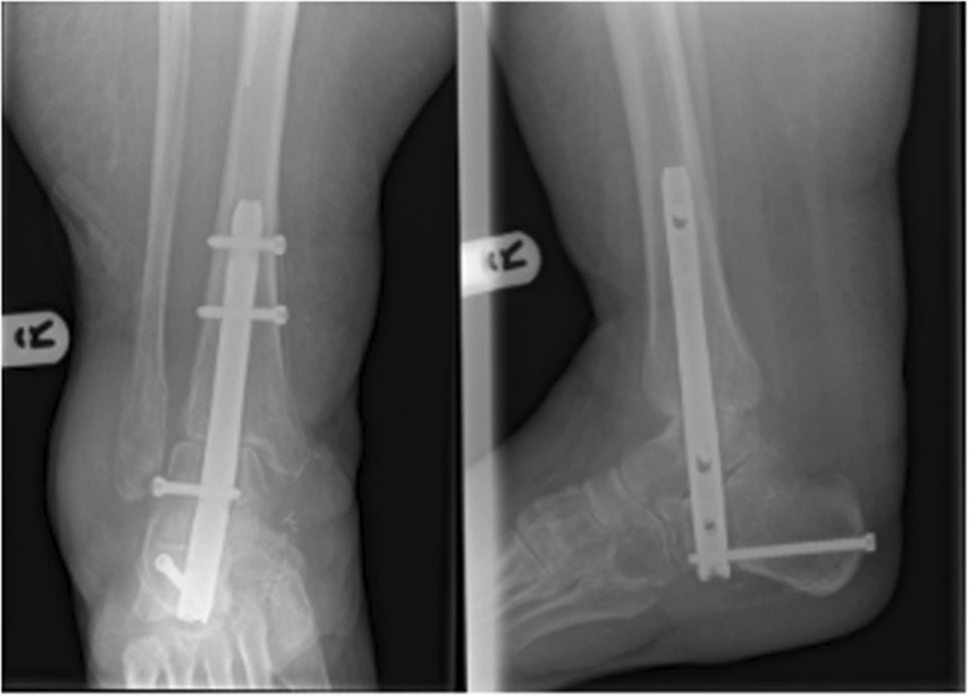
Post-operative radiographs after TCC nailing. Source: Obtained from Ref. 24

Armstrong [24] described 21 ankles in 20 patients over a 5-year period from (July 2011–June 2016). Seven of these patients met our inclusion criteria, as they had a mean age of 89 years (range 82–98). Both high- and low-energy traumas were included. Based on the mechanism of injury and other injured regions we were able to filter the low-energy traumas. One patient aged 83 was excluded because of high-energy trauma. Three different nails were used. All nails were locked proximally and distally (see Fig. 3). All required soft tissue coverage performed by a plastic surgeon.

### Outcomes

In Table 5, an overview of all the different outcome factors is displayed. Mobility, comorbidities, complications, mortality, wound healing, post-op weight-bearing status, and fracture classification are the outcome factors that were observed in four or more studies.

**Table 5:** Outcome factors

Patient characteristics	
Mobility	Meijer, Schray, Shivarathre, O’Daly, Armstrong
Comorbidities	Meijer, Schray, Shivarathre, Armstrong
American Society of Anesthesiologist classification ASA	Schray, Shivarathre
Charlson comorbidity index (CCI)	Schray
Residence	Schray, Armstrong
Pain	O’Daly
Medication	Schray
Karlsson score	Schray
Japanese society of surgery of the foot, ankle–hindfoot scale (JSSF score)	Schray
Risk factors	Shivarathre

Treatment characteristics	
Number of surgeries	Meijer
Re-operations	Schray, Armstrong
Hospitalization days	Meijer, Schray, Armstrong
Outpatients contacts	Meijer
Complications	Meijer, Schray, Shivarathre, O’Daly Lorente, Armstrong
Fixation days	Meijer
Wound healing	Meijer, Schray, Shivarathre, O’Daly
Discharge management	Schray, Armstrong
Mortality	Schray, Shivarathre, Armstrong
Costs	Lorente
Anesthesia during surgery	Shivarathre
Methods of soft tissue coverage	Armstrong
Post-op weight bearing status	O’Daly, Shivarathre, Armstrong, Lorente

Quality of life	
SF-12	Lorente
EQ-5D 3 L	Schray
Barthel Index	Lorente, Schray

Fracture characteristics	
Weber classification/fracture type/fracture pattern	Meijer, Schray, Shivarathre, O’Daly
Anatomical position	Meijer
Radiographic union	Shivarathre, O’Daly
Mechanism of injury	Armstrong
Fracture configuration	Armstrong
Grade of open fracture	Armstrong

#### Post-operative weight-bearing status and mobility

Concerning post-operative weight-bearing status, the patients in the study of Shivarathre et al. [19] had 6 weeks non-weight-bearing below-knee plaster cast. Among 87 patients, 75 patients (86%) returned to pre-injury mobility in 3–6 months. Six patients (9%) who were mobilizing independently before the injury had to use canes and another three patients had to use a walker to aid mobilization. Schray et al. [20] looked at the pre-operative Parker Mobility Score (PMS) which was lower in the group of patients aged 80 and over compared to the patients aged younger than 80 years (*p* = 0.014). The post-operative PMS of patients aged 80 and over was not given. The pre-operative mobility independency is given and a distribution of the ability to walk post-operative. These outcome factors were both given for the patients aged 70–80 as well as the patients aged 80 and over. In this study, only the pre-injury independency is described and the post-operative distribution of ability to walk. However, with these numbers, it is not possible to determine how many patients have returned to their pre-injury mobility. In the study population of Meijer et al. [21], two out of seven patients (29%) returned to their pre-injury mobility. Lorente et al. [22] compared weight-bearing versus non weight-bearing in patients treated with a plaster cast and found no difference between the two groups as regards complications. Moreover, they found a better quality of life in the wb group. In the study of O’Daly et al. [23], all patients commenced full weight-bearing with a walking frame, with assistance as far as required on day one post-operatively. Early weight-bearing is a possibility due to the immediate stability the Gallagher nail provides. Moreover, the threaded screw mechanism at the proximal end effectively prevents proximal migration of the nail. Of the six patients that met our inclusion criteria, four returned to their previous mobility status (66.7%). The majority of the patients of the study of Armstrong et al. [24] were non weight-bearing on the operated ankle for 2 weeks to allow flap healing (range 1–6 weeks). After this, weight-bearing as tolerable was advised. All the patients were mobilized by 2–6 weeks post-operatively (mean 3 weeks). Only one patient of the seven could be discharged to the same residence as pre-injury. There are no data about long-term mobility. Schray et al. [20] and Meijer et al. [21] did not describe the post-operative weight-bearing status of their patients. Lorente et al. [22] did not describe the return of pre-injury mobility.

#### Quality of life

Schray et al. [20] compared patients aged 70–80 and aged 80 and over on quality of life measured through the EQ-5D 3 L, activities in daily life measured through Barthel Index, functional outcome evaluated with the Karlsson score, and the Japanese Society of Surgery of the Foot, Ankle–hindfoot scale (JSSF) score. Patients aged 70–80 had significantly superior results in all four scores (see Table 6). Lorente et al. [22] used the SF-12 and Barthel Index to measure the quality of life. The average quality of life post-operatively measured with the SF-12 and the Barthel Index was significantly higher (*p* < 0.001) in the weight-bearing (wb) group compared to the non-weight-bearing (nwb) on every follow-up moment. The other studies did not use quality of life as an outcome factor.

**Table 6:** Schray et al. age-related outcomes [20]

	Aged > 80 years	Aged < 80 years	*p* value
EQ-5D 3 L	80 ± 20	43 ± 18	< 0.001
Barthel Index	96 ± 6	69 ± 22	< 0.001
Karlsson score	79 ± 20	55 ± 23	0.016
JSSF score	155 ± 30	88 ± 59	< 0.001

#### Complications and wound healing problems

Shivarathre et al. [19] described 77 out of 87 patients (88.5%) showed complete wound healing after 3 weeks. Superficial wound infections occurred in six patients (7%) which were successfully treated with oral or intravenous antibiotics during 1–2 weeks. None of them required further surgery. In four cases (4.6%) deep infection occurred which was treated with intravenous antibiotics. However, all four patients eventually required debridement and removal of hardware after 4–6 weeks, and eventually, no plastic surgery intervention was needed. Statistically significant risk factors associated with wound complications were noted to be diabetes, dementia, smoking, and peripheral vascular disease (*p* < 0.05). For diabetes mellitus, the relative risk ratio (RR) was the highest with 6.6. Schray et al. [20] described that of the post-operative complications, 29% of the study population had non-surgical complications and 20% had surgical complications. A wound healing impairment was found in 10%. Meijer et al. [21] subdivided the complications in surgical and non-surgical. Three patients (33%) suffered from a superficial wound infection, and no patient had a deep wound infection. Lorente et al. [22] found that in the nwb group, a complication rate of 40.5% was registered and in the wb group, this percentage was 51.5%. Statistically, there was no significant difference in complications (Table 7) between the two groups. O’Daly et al. [23] described that no intraoperative complications occurred. No wound complications occurred, despite the poor skin condition at the time of surgery, and all wounds healed within 7 days. Armstrong et al. [24] described the following complications, one re-operation was needed because of flap problems. No re-operations occurred because of implant-related problems and none have undergone amputations. The overall superficial wound infection of all the ankles [21] was 29%. The percentage could not be calculated for the seven patients of 80 years. There were no deep infections.

**Table 7:** Lorente et al. complication numbers [22]

Complication	NWB (*n* = 33)	WB (*n* = 33)
Secondary fracture displacement	3	4
medial pseudo-arthrosis malleolus	2	3
Lateral pseudo-arthrosis malleolus	1	1
Soft tissue problems	6	5
Rescue surgery	3	4
*Total*	*15*	*17*

#### Mortality

Shivarathre et al. [19] described that the 30-day post-operative mortality was 5.4%, 90 day was 8.7%, and the 1 year was 12%. Schray et al. [20] described a 1-year mortality of 10%. The mortality in Armstrong et al. [24] was 14% in the population that met our inclusion criteria and 23% in the population under 80 years of age. The causes of death were not related to the surgery.

## Discussion

To our knowledge, this is the first systematic review describing different kinds of treatments and outcomes specifically for patients aged 80 years and over. In the included studies the following treatments were described for this population; ORIF, transarticular Steinmann pin, plaster cast with or without weight-bearing, Gallagher nail and the TCC nail. Concerning the outcomes; mobility, post-operative weight-bearing status, wound healing and complications seem to be the most used and most relevant outcome factors to assess considering the population.

### Post-operative weight-bearing status and mobility

The various studies show that practitioners are careful with early weight-bearing. However, if we look closely to the results and other literature, this seems not necessary and it could potentially be of great value to implement early weight-bearing in the treatment. Unfortunately most of the literature focus on younger patients or a wider age range than our age group or focus on other kind of lower limb fractures like distal femur fractures like Consigliere, Passias, and Firoozabadi [25–27]. Moreover, emphasizing the need of research concerning early weight-bearing in patient with an ankle fracture in our population. Kalmet et al. [28] published a study protocol of a prospective multicenter comparative cohort study of permissive weight-bearing in trauma patients with fractures of the lower extremities treated surgically. Permissive weight-bearing is an early weight-bearing guided by subjective experiences (e.g., pain, weight-bearing tolerance) of patients and therapists and objective parameters (e.g., temperature of limb, edema). A regime of weight-bearing that would be of value for our population. Early weight-bearing stimulates the patient to be active in daily life. Exercise and early weight-bearing accelerate return to daily activities [30]. Furthermore, elderly patients are often unable to comply with non-weight-bearing instructions [28, 31] and many patients are unable to tolerate prolonged periods of bed rest as they are at risk of medical complications such as pneumonia and pressure sores. The Gallager nail seems to provide the possibility of early weight-bearing due to the immediate stability the device offers. Nevertheless, we tend to be cautious with the conclusions from O’Daly et al. [23] due to the relatively low quality of the study. Other literature concerning the Gallagher nail was not found, therefore, we could not verify their findings.

TCC nailing with flap coverage allows quick return to mobility, limited only by the need for soft tissue protection immediately post-operatively. The soft tissue protection causes a non-weight-bearing period of at least 1 week, which influences the critical mobilizing process of the patient. Nevertheless, this technique still allows early weight-bearing which is backed up by the literature. Georgiannos et al. [5] compared ORIF (*n* = 44) with TCC (*n* = 43) for a population with an ankle fracture with a mean age of 78 years (range 70–97). No division between under 80 years and above 80 year was given, therefore, the study was excluded from our review. They found that TCC had significantly less complications (TCC 8.1%, ORIF 33.3%, *p* < 0.05) and that the length of hospital days was significantly shorter for TCC (TCC 5.2, ORIF 8.4, *p* < 0.001). Furthermore, Georgiannos et al. [5] found that 18 patients of the TCC group returned to their pre-injury mobility status (81.8%) while 4 patients declined one level of the mobility scale (18.2%). In the ORIF group, 17 patients remained at the same mobility level (73.9%) and 7 patients declined one level (26.1%). They concluded that TCC nailing is a safe and effective method of treatment of unstable ankle fractures in the elderly because it has a low risk of complications and restores function and mobility and immediate return to full weight-bearing ambulation. Similar results, except for the mobility results, can be observed in the results of Armstrong et al. [24].

### Quality of life

Quality of life was only described in two of the included studies. The average life expectancy of women has a 50% probability to increase beyond 90 by 2030 [9]. In our opinion, quality of life is a necessary outcome factor to add to the usually used outcome factors, considering the life expectancy of this age group. Despite this increase in life expectancy, this is still an age group with a limited life expectancy. Therefore, functional outcome may be clinically more relevant for this population than radiological/anatomical outcome. However, there are limited studies on this subject that use quality of life as an outcome factor.

### Complications and wound healing problems

From the included studies is seen that weight-bearing does not increase the number of complications compared to a non-weight-bearing regime. A phenomenon that is as well seen in the literature [33]. However, this was a study population with a younger age group. Lynde et al. [29] report on a cohort of 216 patients with a mean age of 69.9 years (range 60–95). They found that surgical treatment of unstable ankle fractures in the elderly is fairly predictable with an acceptable complication rate. The complication rates were higher in patients with diabetes mellitus. However, patients who were allowed to walk within the first 2 weeks post-operatively did not experience higher rates of hardware failure; results that are in line with those of Shivarathre et al. [19].

Lorente et al. [22] described the use of plaster cast reported no significant difference in complications between the nwb and the wb groups, this is as well an effect of small number of complications. The complication rates of, respectively, 40.5 and 51.5% for the nwb and wb groups are not further explained in the study. Lorente et al. [22] only focus on whether there is a difference between the two groups and not especially the complication rate itself. However, it are relatively high complication rates if they are compared to the complications rates of other studies. Nevertheless, this study had a very specific study population with prone elderly which where note fit for surgery, which may explain the relatively high complication rate.

The complications rates described by Armstrong et al. [24] are higher than described in other literature suggest like Al-Nammari et al. [4]. They describe 48 frail elderly with a mean age of 82 years (61–96) treated with a TCC nail and the complications included superficial infection (4%, two of 48) and deep infection (2%, one of 48). The literature suggest that the TCC nail is even a considerable treatment for patients with diabetes, probably since it is a minimal invasive technique [32]. The results shown in Armstrong are supported by Al-Nammari et al. [4]. According to Al-Nammari et al. [4] TCC nailing is an excellent treatment for the frail elderly since it allows the patient to mobilize immediately and minimizes the risk of bone or wound problems.

### Mortality

The mortality was described for the following treatments: ORIF and the TCC nail. For both a comparable mortality percentage was found. However, unfortunately the mortality was not described for all the different kind of treatments. A mortality percentage of all the studies would have improved the value of the overall comparison of the different treatments. Nevertheless, age or ASA-classification is probably the major predictor of mortality, not the choice of treatment of the ankle fracture.

At the time of the revision (May 2021) we did an update of the research. This revealed 185 articles with in the end one article with substantial extra information. Gil et al. [34] performed a retrospective cohort study with the hypothesis that mortality and complications of ORIF in patients who are 65–79 years of age are equivalent to ORIF in patients who are 80–89 years of age. The study included 2353 ankle fractures: 1877 were among 65–79 and 476 were among 80 or older. Results showed a 3.2 fold significantly higher risk of 30 day mortality in the 80–89 years age group compared to the 65–79 years old group (1.47 versus 0.48%). There was also a higher morbidity incidence in the 80 years and older group compared to the younger geriatric group, reflecting 30 days re-operation rate, perioperative blood transfusion rate, and urinary tract infection rates were significantly higher in the older group. After controlling for the ASA class, patients aged 80 or older who underwent ORIF of their ankle fractures no longer had a significantly higher mortality and no longer had rate of complications in comparison to the 65–79 years old age group. This suggests that the increased mortality and complication rates seen in this cohort reflects the higher burden of systemic disease. The results indicate that it is important to consider surgical treatment options for older (i.e., 80–89 years old) geriatric ankle fractures because mortality and morbidity rates are comparable with the 65–79 years old group when medical comorbidities (i.e., the ASA class) are taken into consideration.

Other operations and conservative methods are mentioned in the literature as treatments for ankle fractures. However, no specific data about these treatments are known for patients aged 80 and over. Other treatments are, for example, external fixation as described in Meijer et al. and the Fibular nail [35, 36], or invasive techniques such as rush nails, intramedullary screws, fibula-rod [37].

This systematic review has several limitations. The review included studies with a relatively low level of evidence. Unfortunately, to our knowledge, no randomized controlled trial has taken place on this subject. Due to the limited available evidence, case series were included. However, a case series is a study design that is prone to bias which emphasizes the importance of a critical assessment. Most of the studies only describe one treatment. Therefore, we have to be cautious with comparing and interpreting the results and drawing conclusions. We did not include case reports due to their low level of evidence. Therefore, we could have missed some underreported treatments. Furthermore, there is still not a sufficient search filter for this specific age group [11]. Hence, a well-considered combination of terms has to be made as precise as possible.

The most important strength of this systematic review is the age restriction. The study only focused on patients aged over 80 years, which is a neglected population in the literature. Most studies on this subject have a larger age range despite the different patient characteristics between patients aged 80 and younger-aged patients. Since we have a rapidly aging population, studies for this specific age group are needed.

There are still many unanswered questions about ankle fractures in patients aged 80 and over. Subjects that warrant more attention are the treatments that are still not well documented for this age group. Other interesting questions are whether there is a relation between, for example, duration of fixation and post-operative mobility or length of hospital stay and final outcome. Another subject that needs attention is the possibility of early weight-bearing after ORIF and other surgical interventions and the associated complication rates for this specific age group. Furthermore, studies that compare ORIF and minimal invasive surgery in light of complications would be helpful in the treatment decision making. Future studies on the current topic, especially high-quality evidence studies, are, therefore, recommended.

## Conclusion

We hoped to find evidence to determine the best treatment. However, due to a lack of literature for this population, this was not possible. What we could conclude with the available literature is that surgical treatment, both invasive and minimal invasive, seems to be a safe treatment for the elderly. We conclude that ORIF with plaster cast and permissive weight-bearing should be considered for this population since it seems to be a safe possibility for a majority of the relatively healthy patients aged 80 and over without an increase in the complication rate. Nevertheless this approach holds the risk of implant failure. Additionally elderly patients may fail to comply with partial weight-bearing regimes. This should be taken in consideration in the treatment choice. We would recommend considering TCC nailing, especially in the more complication prone elderly patients aged 80 and especially these with diabetes or peripheral vascular disease, since TCC is a minimal invasive technique and early weight-bearing is possible. Steinman pins and Gallagher nails cannot be recommended because of lack of evidence. When surgery is contra-indicated and a plaster cast is the choice of treatment, early weight-bearing seems to have a positive influence on the outcome in the very old patient. The authors recommend to use post-operative mobility, quality of life, and post-operative weight-bearing status as important outcome factors in this population, rather than radiological or anatomical results in future research.

## Appendix

### Appendix 1: Search strings

#### Appendix 1.1 Search string Pubmed

**Table:**

Search	Query	Results
#11	Search: #10 Filters: from 2010 -2020	751
#10	Search: ((("Ankle Fractures"[Mesh]) OR (("Fractures, Bone"[Mesh:noexp]) AND ("Ankle"[Mesh] OR "Ankle Joint"[Mesh]))) OR ((ankle*[Title/Abstract] OR malleol*[tiab] OR talus[tiab] OR talar[tiab] OR regio tarsal*[tiab] OR tarsus[tiab]) AND (fracture*[tiab] OR rupture*))) AND ((("Aged, 80 and over"[Mesh]) OR (Frail Elderly[Mesh])) OR (“aged 80”[tiab] OR 80 year*[tiab] OR 80 year*[tiab] OR Centenarian*[tiab] OR eighty year*[tiab] OR Elderly[Title/Abstract] OR Fragility[tiab] OR frail*[tiab] OR Geriatric*[tiab] OR Octogenarian*[tiab] OR Oldest-old*[tiab] OR onagenarian*[tiab] OR Senior*[Title/Abstract] OR Supercentenarian*[tiab] OR very elderly[tiab] OR very old[tiab]))	1,215
#9	Search: (("Aged, 80 and over"[Mesh]) OR (Frail Elderly[Mesh])) OR (“aged 80”[tiab] OR 80 year*[tiab] OR 80 year*[tiab] OR Centenarian*[tiab] OR eighty year*[tiab] OR Elderly[Title/Abstract] OR Fragility[tiab] OR frail*[tiab] OR Geriatric*[tiab] OR Octogenarian*[tiab] OR Oldest-old*[tiab] OR onagenarian*[tiab] OR Senior*[Title/Abstract] OR Supercentenarian*[tiab] OR very elderly[tiab] OR very old[tiab])	1,174,998
#8	Search: “aged 80”[tiab] OR 80 year*[tiab] OR 80 year*[tiab] OR Centenarian*[tiab] OR eighty year*[tiab] OR Elderly[Title/Abstract] OR Fragility[tiab] OR frail*[tiab] OR Geriatric*[tiab] OR Octogenarian*[tiab] OR Oldest-old*[tiab] OR onagenarian*[tiab] OR Senior*[Title/Abstract] OR Supercentenarian*[tiab] OR very elderly[tiab] OR very old[tiab]	385,541
#7	Search: Frail Elderly[Mesh]	11,330
#6	Search: "Aged, 80 and over"[Mesh]	906,681
#5	Search: (("Ankle Fractures"[Mesh]) OR (("Fractures, Bone"[Mesh:noexp]) AND ("Ankle"[Mesh] OR "Ankle Joint"[Mesh]))) OR ((ankle*[Title/Abstract] OR malleol*[tiab] OR talus[tiab] OR talar[tiab] OR regio tarsal*[tiab] OR tarsus[tiab]) AND (fracture*[tiab] OR rupture*))	11,403
#4	Search: (ankle*[Title/Abstract] OR malleol*[tiab] OR talus[tiab] OR talar[tiab] OR regio tarsal*[tiab] OR tarsus[tiab]) AND (fracture*[tiab] OR rupture*)	10,869
#3	Search: ("Ankle Fractures"[Mesh]) OR (("Fractures, Bone"[Mesh:noexp]) AND ("Ankle"[Mesh] OR "Ankle Joint"[Mesh]))	2,568
#2	Search: ("Fractures, Bone"[Mesh:noexp]) AND ("Ankle"[Mesh] OR "Ankle Joint"[Mesh])	1,254
#1	Search: "Ankle Fractures"[Mesh]	1,463

#### Appendix 1.2 Search string Embase

**Table:**

Search	Query	Results
5	4 Specific year range: 2010–2020	718
4	((Fracture/ and Ankle/) or Ankle fracture/ or ((ankle* or malleol* or talus or talar or regio tarsal* or tarsus) and (fracture* or rupture*)).ti,ab,kw.) and (Very elderly/ or Frail elderly/ or (aged 80 or 80 year* or 80 year* or Centenarian* or eighty year* or Elderly or Fragility or frail* or Geriatric* or Octogenarian* or Oldest-old* or nagenarian* or Senior* or Supercentenarian* or very elderly or very old).ti,ab,kw.)	912
3	1 and 2	912
2	Very elderly/ or Frail elderly/ or (aged 80 or 80 year* or 80 year* or Centenarian* or eighty year* or Elderly or Fragility or frail* or Geriatric* or Octogenarian* or Oldest-old* or nagenarian* or Senior* or Supercentenarian* or very elderly or very old).ti,ab,kw	695,072
1	(Fracture/ and Ankle/) or Ankle fracture/ or ((ankle* or malleol* or talus or talar or regio tarsal* or tarsus) and (fracture* or rupture*)).ti,ab,kw	14,357

#### Appendix 1.3 Search string Cochrane

**Table:**

ID	Search	Hits
#1	MeSH descriptor: [Ankle Fractures] explode all trees	145
#2	MeSH descriptor: [Fractures, Bone] explode all trees	5982
#3	MeSH descriptor: [Ankle] explode all trees	488
#4	MeSH descriptor: [Ankle Joint] explode all trees	687
#5	(ankle* OR malleol* OR talus OR talar OR regio tarsal* OR tarsus):ti,ab,kw AND (fracture* OR rupture*):ti,ab,kw	1264
#6	MeSH descriptor: [Aged, 80 and over] this term only	51,911
#7	MeSH descriptor: [Frail Elderly] this term only	710
#8	(“aged 80” OR “80 year*” OR 80 year* OR Centenarian* OR “eighty year*” OR Elderly OR Fragility OR frail* OR Geriatric* OR Octogenarian* OR Oldest-old* OR onagenarian* OR Senior* OR Supercentenarian* OR “very elderly” OR “very old”):ti,ab,kw	102,879
#9	(#3 OR #4) AND #2	47
#10	#1 OR #5 OR #9	1264
#11	{OR #6-#8}	102,879
#12	#10 AND #11	129
#13	#12, year filter 2010–2020	97

#### Appendix 1.4 Search string CINAHL

**Table:**

#	Query	Results
S8	S7 year Filter 2010–2020	488
S7	(S4 OR S5) AND (S3)	655
S6	S4 OR S5	435,757
S5	(“aged 80” OR “80 year*” OR Centenarian* OR “eighty year*” OR Elderly OR Fragility OR frail* OR Geriatric* OR Octogenarian* OR Oldest-old* OR onagenarian* OR Senior* OR Supercentenarian* OR “very elderly” OR “very old”)	435,757
S4	MH “Aged, 80 and over” OR MH “Frail Elderly”	314,824
S3	S1 OR S2	6,011
S2	(ankle* OR malleol* OR malleol* OR talus OR talar OR regio tarsal* OR tarsus) AND (fracture* OR rupture*)	6,011
S1	(MH “Ankle Fractures” OR MH Fractures) AND (MH Ankle OR MH “Ankle joint”)	533

## Abbreviations

ORIF: Open reduction and internal fixation
TCC: Tibiotalocalcaneal
